# Acknowledgment to Reviewers of *Non-Coding RNA* in 2021

**DOI:** 10.3390/ncrna8010014

**Published:** 2022-01-29

**Authors:** Non-Coding RNA Editorial Office

**Affiliations:** MDPI AG, St. Alban-Anlage 66, 4052 Basel, Switzerland

Rigorous peer-reviews are the basis of high-quality academic publishing. Thanks to the great efforts of our reviewers, *Non-Coding RNA* was able to maintain its standards for the high quality of its published papers. Thanks to the contribution of our reviewers, in 2021, the median time to first decision was 15 days and the median time to publication was 37 days. The editors would like to extend their gratitude and recognition to the following reviewers for their precious time and dedication, regardless of whether the papers they reviewed were finally published:
Abhishek DeyLei ShiAlan DombkowskiLeonard LipovichAlessandro SammarcoLetizia PittoAlex De LencastreLin HuangAlexey NikulinLorenzo FarinaAlfonso M. CayotaLuca AgnelliAndré GerberLuca Lo PiccoloAndrea CaporaliLucia NatarelliAngel Vizoso-VázquezLuke SelthAnisha GuptaMai HazekawaAnna RiesterMarco GaviraghiAntoine M. DujonMargarida Gama-CarvalhoAntonio MusioMaria DuhagonAria BaniahmadMaria Elizabeth Rossi Da SilvaArijita SarkarMaria HondeleArjen E. Van’t HofMaria SundvallArturo López CastelMarina CristoderoAssam El-OstaMartin SztachoBabu V. SajeshMartina KirchnerBei ChengMartina RossiBodhisattwa BanerjeeMatthias S. LeisegangByron BaronMatus SykoraCameron BrackenMd. Motiar RahmanCecilia BattistelliMichael TellierCelia Fernández RubioMichele De BortoliCesar Lopez-CamarilloMohammed L. AbbaCharles Bou-NaderMunekazu YamakuchiChristine HappelNatalia SimionescuChristopher HellenNicholas DelihasChristos K. KontosNicolas CasadeiChristos PapaneophytouNicoletta BianchiClaudia KutterNithyananda ThorenoorCristian TaccioliOliver MühlemannDaniela GradiaOvidiu SirbuDavid C. ZappullaPablo SmircichDavid LeavesleyPanagiotis AlexiouDeepanjan PaulPaola OstanoEdoardo BertoliniPatricia Mirella ScarduaElena López-JiménezPavel IvanovErica ScottPeter BaiEstanis NavarroPraveen AranyEsteban OrellanaPrzemysław NucFederico Dajas-BailadorQinyu SunFrancisco J. EnguitaRajneesh SrivastavaFranck MartinRobert WeinzierlGaetano SantulliRocio T. Martinez NunezGianpiero Di LevaRoland KlassenGiulia MatacchioneRui FuGiuseppe PalmieriRussell HamiltonGiuseppina E. GriecoRyuya FukunagaGrasiele SausenSabyasachi DashGunter MeisterSamuel Pushparaj Robert JeyasinghHelen VincentSamuela PasqualiHenrik NielsenSatya KotaHiroji AibaSébastien PfefferHisashi MeraShahrokh GhobadlooHua-Sheng ChiuShizuka UchidaHui LiShu-Ling TzengIlaria GuerrieroSilvia LeeIzabella Slezak-ProchazkaSimon ConnJan NovákSimon RaynerJarmila HnilicováSimona ZaamiJens HahneStefan Ernst SeemannJianhua WangStefano CagninJiao Jiao LiStephan BernhartJijun HuangStephan HamperlJoanna Sztuba-SolinskaSuba RajendrenJoanna WilliamsSumit MukherjeeJoe ChihadeTadashi YoshidaJosé AndradeTanja KunejJuan José González-PlazaVasiliki TsigkouKatherine McJunkinXucheng HouKathrin TyryshkinYork MarahrensKausik ChakrabartiYoshihiro TaguchiLasse Lindahl



